# Advancing Person-Centred Care Through Palliative Care WA’s ACP Workshops: The Longer-Term Impact

**DOI:** 10.1177/00469580251371891

**Published:** 2025-09-28

**Authors:** Gertrude G. Phiri, Emma Gunner, Philip Bafile, Davina Porock

**Affiliations:** 1 Edith Cowan University, Joondalup, WA, Australia; 2 University of Western Australia, Perth, WA, Australia; 3 Mahasarakham University, Maha Sarakham, Thailand

**Keywords:** palliative care, end-of-life care, decision-making, advance care planning, advance health directive

## Abstract

Planning for end-of-life and associated decision-making is one of global significance as it impacts the quality of care in palliative care settings, including ensuring that personal preferences are honoured. It is a priority for the Australian health system to use Advance Care Planning (ACP) as it is an effective tool for individuals and their families to make their personal preferences known and implemented. The aim of this project was to evaluate the longer-term impact of the Palliative Care Western Australia (PCWA) ACP workshops. Semi-structured, open-ended questions were used to collect data from 21 participants. Reflexive thematic analysis was used to analyse the data. Three themes emerged: (1) Finding the words, (2) Finding the people and (3) Finding the way. The workshops enabled individuals to complete their Advance Health Directives (AHD) correctly, substitute decision-makers to gain confidence in enacting the AHD for their loved ones, and healthcare professionals to provide seamless, person-centred care.

## Introduction

The importance of planning for the end-of-life (EOL) and associated decision-making is one of national^1^) and international.^2^ It is clear from previous research that planning for care and specifically aiding decision-making near the EOL has a significant impact on the quality-of-care provision, including ensuring that personal preferences are honoured.^3,4^ It is a priority therefore for the Australian health system that Advance Care Planning (ACP) be used as an effective tool for individuals and their families to have their personal choices and preferences known and implemented.^5^

Palliative Care Western Australia (PCWA) has led the way in educating Western Australians from all backgrounds in understanding ACP and supporting individuals and carers to document those plans appropriately. PCWA is a not-for-profit peak body in Western Australia (WA) representing palliative care providers and professionals whose aim is quality of life for all. This is achieved by raising awareness and understanding of palliative care in the community and improving access to palliative care services. Secondly, it encourages, recognises, and rewards excellence in palliative care delivery, and thirdly, acts as the bridge between the government and the palliative care sector.^6^ The major focus of the community education strategy over the last 10 years has been on delivering free interactive community workshops throughout metropolitan and regional WA. ACP workshops were promoted through the Local Government Authorities and newspapers. The workshop agenda included what was involved in ACP, exploring individual’s priorities for EOL, having EOL conversations, what palliative care was all about and how to complete the Advance Health Directive (AHD) and Enduring Power of Guardianship (EPG). Workshop participants were offered the ACP resource pack, the same could also be accessed online. Following 2 h of face-to-face group interactive meetings facilitated by a qualified workshop facilitator, participants were offered (and many took the offer) a 1-h, one-on-one follow-up visit by PCWA ACP dedicated Counsellors to assist with accurately completing the AHD document. Previous evaluations of the ACP workshops have shown the powerful impact of these sessions. They have revealed statistically significant rates of improvement in understanding ACPs and completion of ACP documentation.^7^ Furthermore, there are many satisfied participants who have found the workshops invaluable, and there is a growing demand for participating in the PCWA ACP workshops (Workshops).^8^

It is known that patients and carers gain knowledge and understanding from the workshops.^8^ Many, if not most participants, translate that knowledge into formal ACP documentation which occurs up to several months after attending a workshop.^7^ The question which remains is that: with the documents completed, utilising the support of workshops and Counsellors, and with confidence built in EOL decision-making and advocacy, what happens when the time comes to enact the ACPs?

The Australian Institute of Health and Welfare (AIHW) estimates that nearly half (47%) of Australians live with chronic conditions. Four out of every 5 persons aged over 65 have 1 or more chronic conditions and 20% of the total Australian population live with 2 or more chronic diseases.^9^ Furthermore, 70% of all Australians say they wish to die at home, yet less than 15% of all deaths in Australia occur at home.^10^ For those wanting to die at home or to have their wishes respected in hospital, hospice, or residential aged care, the AHD is the most secure way to guide their care according to personal preferences. The workshops are aimed at creating awareness and empowering individuals to make their EOL care preferences and decisions known and documented. Additionally, they encourage the dissemination of this information, not only to loved ones, but also to health professionals and carers so they can be cared for according to their wishes. This is the longer-term impact of the workshops on participants’ lives going forward.

### Evaluation Question

What is the longer-term impact of Palliative Care WA Advance Care Planning Workshops?

## Methods

This qualitative evaluation of the longer-term impact of the PCWA ACP workshops was conducted in Western Australia from April to October 2024. In this study, longer-term is defined as how information gained from workshops impacted participants’ lives going forward. The study followed the appropriate EQUATOR guidelines^11^ designed for qualitative inquiries.

### Ethics Approval

This study received approval from an Institutional Human Research Ethics Committee in April 2024.

### Sampling

To evaluate the longer-term impact, potential participants were emailed from the list of attendees at the workshops between 2018 and 2024. PCWA contacted attendees with information about the study, inviting potential participants to contact the evaluation team directly. Information about the study was also published in the regular PCWA e-newsletter with contact information for the research team. From the responses, we recruited the following 3 groups:

a. Individuals who attended the workshop/s for personal benefit.
b. Family members or significant others who enacted an AHD on behalf of a loved one.
c. Healthcare professionals who attended a workshop and had also cared for patients and/or family members with and without an AHD.

### Data Collection

Qualitative telephone and online interviews were undertaken by a female professor (DP), experienced qualitative research and 2 female PhD candidates (GP and EG). Each interview took between 15 and 30 min. Interviews conducted online were transcribed by Microsoft Teams and then corrected by the interviewer. Each participant attended the interview un-accompanied. Telephone interviews were audio recorded and transcribed verbatim using NVivo software. Three telephone interviews were not audio recorded, and the interviewer made detailed field notes during and after the interview. Some participants also provided written information which was included as data. Basic demographic information was gathered for all 3 participant groups during the interview. All data were entered and managed using NVivo software. Participants’ interview questions were arranged according to each group; individuals, family/substitute decision-makers, and healthcare professionals.

For individual participants attending the workshops for personal benefit, the interview topics included:

- General impressions of the workshop and its usefulness and adequacy in preparing for EOL decision-making and care.
- The feeling of confidence to put the concepts and knowledge gained into practice.

For family members/substitute decision-makers the topics included:

- Telling the story of using ACP as part of the care of the family member.
- Helpfulness of attending the workshop in making decisions on behalf of a family member.
- Confidence in enacting the ACP as a substitute decision-maker.
- What would or could be done differently if given another opportunity to make decisions on behalf of someone?

For healthcare professionals and care staff associated with EOL care the topics included:

- Describing the care of the person with the ACP.
- Experience of caring for someone who had documented/ made known their decisions, preferences and values (compared with those who did not).

The interview guides were pilot tested on the first 3 participants, one from each category and reviewed and did not require modification. Data saturation^12^ was reached after conducting 16 interviews. Nevertheless, 5 additional interviews were conducted to ensure clarity of the themes.

### Data Analysis

Two types of analysis were planned. The first line of analysis took a conventional approach to the coding and analysis of the interviews, permitting the identification of concepts and themes across all participants. Each transcript was coded by 2 team members before discussing and reaching consensus at regular 4-member team DP, GP, EG and PB (a male Masters student) meetings. A reflexive thematic approach to the analysis was used, as described by Braun and Clarke.^13,14^

In the second line of analysis, we aimed to create case studies to assess the longer-term impact of AHDs and the experience of their influence during episodes of care towards the EOL for all involved, including the substitute decision-maker, various family members and connected health professionals and carers. It was hoped this would provide a unique view of how the longer-term outcomes of the workshops had developed. However, despite referrals from substitute decision-makers to the General Practitioners (GPs) who had provided care at the EOL, no GPs returned the phone call or email invitations. The reason for non-response is not known. With the data collected, the second analysis approach was adjusted to reconstruct the impact stories from 2 perspectives: the substitute decision-maker, and the health professional.

Both types of analysis followed a rigorous process to minimise bias and ensure all voices were heard from the data. Following the transcription and cleaning, each interview transcript was read by all members of the evaluation team. Line-by-line coding was conducted for each transcript by 2 team members and then discussed at the regular team meetings. Identification of clusters of codes and development of themes was achieved through discussion at meetings.

### Recruitment

**Figure 1.**
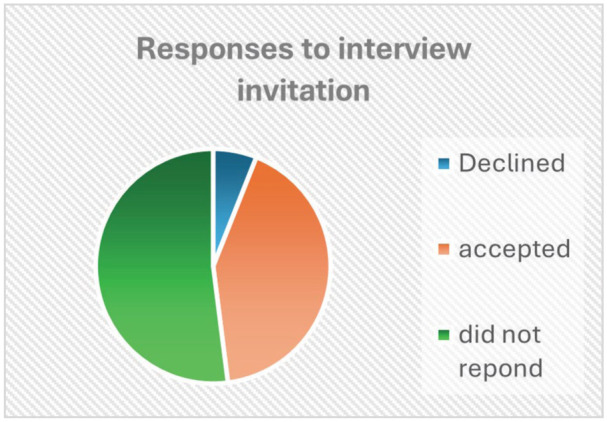
Response to invitation.

**Figure 2.**
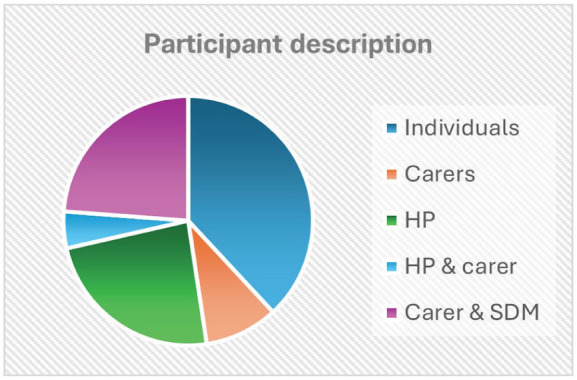
Participant description.

Invitations to participate in the workshop evaluation were sent by PCWA to former attendees of the workshops. A total of 1256 emails were sent with 52 bounce-backs. Sixty individuals called by phone, 2 responders agreed to participate, and they were asked to contact the evaluation team, 30 of these individuals either declined or had a phone number that was no longer in service. A voicemail and/or text message was left for the remaining 28. Invitations were also included in 3 mail-outs to the PCWA e-news list of 1336 people with 6 bounce-backs. Would-be participants were advised to contact the evaluation team directly to express their interest. Following PCWA’s invitation, 57 potential participants contacted the evaluation team. Information and consent forms were sent to the 57 by email and post. Twenty-one signed consents were received, 3 decided not to participate after reading the information letter, and 26 did not respond. Figure 1 demonstrates the distribution of responses and Figure 2 provides the description of participants.

Table 1 further illustrates the limited information we took on participants’ background information.

**Table 1.** Participant Profiles.

Participant	Participant profile	Category	Sex	Age
1	Marriage Celebrant, attended workshop	I	M	70
2	HP, attended workshop	HP	F	61
3	Carer, widower – Carer for and enacted late wife’s AHD.	C/SDM	M	75
4	Individual, attended workshop but has not completed AHD.	I	F	67
5	Individual and Carer – attended workshop: enacted a friend’s (neighbour) AHD.	I/SDM	F	78
6	Individual, attended workshop with husband. Has completed AHD.	I	F	78
7	Individual, attended workshop, has not completed AHD, concerned about making documents more user friendly, language and layout. She is neurodiverse.	I	F	44
8	Carer, widow, enacted late husband’s AHD, has completed AHD, delivered a speech on importance of having an AHD in place to WA parliament in 2023	C/SDM	F	75
9	Individual, attended workshop, has completed an AHD.	I	F	83
10	Healthcare professional and Carer for parents, attended workshop and attended workshop with parents and helped parents complete AHD.	HP/C	F	56
11	Individual, attended workshop with wife, lives in a retirement village, organises PCWA ACP workshops for village residents and has completed AHD.	I	M	83
12	Individual, attended workshop, completed AHD. She cared for ex-husband before he died, before introduction of AHDs.	I/C	F	70
13	Individual, attended w/shop with wife; has completed AHDs.	I	M	73
14	Individual, attended workshop, has filled out the AHD, waiting to discuss with preferred decision-maker before signing.	I	F	77
15	Carer, cared for husband during his EOL; he did not have AHD in place. .	C	F	79
16	Healthcare Professional, attended workshop	HP	F	58
17	Healthcare Professional, attended workshop to gain knowledge on AHD for conversations with residents.	HP	F	45
18	Healthcare Professionals, attended workshop to gain knowledge as she has conversations with clients about completing AHD	HP	F	54
19	Individual/Carer, attended workshop, has not completed AHD. Cared for her mum with AHD in the USA.	I/C	F	60
20	Individual/Carer, attended workshop, has AHD, cared and enacted brother’s AHD.	C/SDM	F	61
21	Healthcare Professional, attended workshop as HP.	HP	F	45

*Note.* I = individual; HP = health professional; C = carer; SDM = substitute decision-maker.

Individuals who attended the workshop/s for personal benefit and significant others who cared or enacted an AHD on behalf of a loved one, were generally older compared to healthcare professionals. Only 1 participant was from culturally and linguistically diverse (CaLD) background.

### Findings

Previous evaluations of the workshops found that workshops were beneficial in assisting individuals to document their EOL preferences in the immediate and medium term. This current evaluation focused on the longer-term impact of the workshops. Although the workshops have been conducted for over a decade, there was still an element of needing information about ACP. Some themes cut across all 3 participant groups. These themes related to the need for information and the significance of attending a workshop. The themes identified were: (1) Finding words, (2) Finding people and (3) Finding the way.

## Themes

### Finding the Words

#### Individuals

The first theme that emerged came from all participant groups who had attended an ACP workshop. Individuals, substitute decision-makers and healthcare professionals found it difficult to talk about issues related to ACP and preparing an AHD. Finding the words to convey their preferences within the AHD was a challenge to individuals as they prepared their documentation. They often found that the words they used to indicate their preferences when completing the AHD were insufficient. Finding the words to start conversations with family and the GP was something the workshop was beneficial for. Before attending the workshop, participants were unaware of what words to use in ACP conversations, they just didn’t know how to talk about what they wanted. For instance, individuals stated that they did not want to ‘be a vegetable’ but did not know the words to write down on the form. The following examples from individuals who attended a workshop illustrate the point.

We wanted to find a way to say, if there was a major problem we want the pain to be relieved entirely even if that shortened our lives. Finding the right way to say things was really difficult. (P13)

Well, if I’m going to be a vegetable, I don’t want to be resuscitated. But you can’t actually write that in a document. So how do you say that and get the meaning across, but be clear enough for other people to make proper decisions? (P11)

One participant self-identified as neurodivergent, stated that they had attended 3 workshops and found it very helpful on each occasion. However, they had been unable to complete the AHD even with one-on-one support because they felt overwhelmed by the amount of written information. They suggested that a video could be made to record preferences and decisions as an official alternative to a written document. Finding the words for this participant meant finding a different way to share their decisions.

### Substitute Decision-Makers

Substitute decision-makers had concerns that they could misinterpret the message in the AHD which would lead to not fulfilling the individual’s preferences. Therefore, it was vital to get the right words that would accurately convey the preferences.

He [her brother] lived independently, but he would not want people to call an ambulance because he was really worried about the action they would take. When he went on a trip . . ., he got a tattoo on his chest that said ‘do not resuscitate’ because he wanted it to be crystal clear to people. I think having had lots of conversations with him, if we just walked in and we’re just dealing with a piece of paper, it would be much harder than if you’ve had lots of conversations (P20)

### Healthcare Professionals

Healthcare professionals, whose work involved helping their patients understand the AHD document and how to complete it, found that they needed to know appropriate words to converse with their patients. The workshops provided them with what was required to support patients who were receiving palliative/EOL care.

. . . it was from having attended the workshop that gave us the conversation, gave us the narrative to actually realise, that there was a discussion to be had. (P10)

I learnt a few of those [words] on how to get the conversations with patients. Yes, that [ACP workshop] helped me. (P 21)

### Finding the People

“Finding the people” describes the problems associated with discussing plans with substitute decision-makers and how attending the workshop helped. In addition to finding the words to clearly and accurately communicate their preferences on the AHD, there was an element of finding people to entrust with the responsibility of decision-making on their behalf when that was needed.

Because the documents themselves talk about so many things that you would never have thought of, and the workshops prepare you for filling in the documents. Then you realise it’s no good just to say, “Oh the kids know what I want”, because they don’t and really, this whole process is important to make sure that you can really have a conversation with the kids. (P 11)

When people consented to become a substitute decision-makers, they wanted to be sure they knew what the individual’s preferences were. The workshop gave them the confidence they felt they needed to ensure they made decisions that honoured the individual’s preferences.

. . .and so, when you are making a decision, you know, in the instance of my brother where we absolutely knew he didn’t want to be kept alive for the sake of keeping alive. But you just have to be confident because you are dealing with someone’s life or death. (P20)

I need to know what she wants now – you can’t un-revive someone if you do the wrong thing. (P13)

One individual felt that blood relatives were not always the best people to entrust future EOL decision-making responsibilities. At times, they were friends or people from a community group such as a church, as illustrated by this participant.

My next step is to go to church, . . . I’m hoping to sit down with them and ask them to take over the care of me [make decisions]. And they know what I want. (P14)

The workshops helped participants think about the details and gave them the words and confidence to talk to the people they chose to speak on their behalf. This newfound confidence enabled them to find a way to discuss advance care planning. They also gained new ideas about how to communicate with their families through interactions with other workshop participants.

### Family Relationships

Substitute decision-makers reported that the workshops gave them skills as decision-makers to carry out their responsibilities. They acknowledged that sometimes, family members may not agree with decisions made on behalf of the individual, but the skills acquired at the workshops gave them confidence to advocate for the individual.

There’ll be a family member who’s not accepting of what’s happening. . . in the majority, yes, the conversations can be hard. PCWA has helped in skilling me to handle such situations and it’s very helpful (P10)

Healthcare professionals also reported having difficulties in providing care when there was no substitute decision-maker appointed and no AHD in place, compared to when there was a completed AHD.

If that discussion hasn’t occurred . . . it is very difficult. . . if they want to die at home, which most of our clients do, and we know that and we’re very clear [through the AHD] on that, then we can do our best to make that happen. (P18)

Participants recognised the potential for family disagreements because of decisions made by a substitute decision-maker. The workshops highlighted the importance of having conversations with their family members. Others recalled negative experiences resulting from not having conversations with family members about their preferences and felt that an AHD if not discussed, had the potential to fracture familial relationships.

And I guess the third bit is making sure you then have the conversations with family. There are always circumstances outside of the control or situations so, they need an understanding . . .to avoid family conflicts. (P20)

### Finding the Way

The impact of attending the workshops was far-reaching in communities where participants lived. They took every opportunity to talk to people around them about how the workshops helped them learn and understand the value of a well-documented AHD. It was evident that attending the workshops resulted in something that became an ongoing conversation for participants. Once their voice had been strengthened by participating in the workshops and, realising the benefits of completing an AHD, one could not help but, advocate for it by talking to family, friends, neighbours and even in parliament.

My name is . . . and I feel it is important to share my experiences, as regards Palliative Care and how we need to share our wishes, about our health care and final moments. (part of speech delivered in Parliament) (P 8)

Healthcare professionals were particularly interested in completed AHDs because they were guided on how to care for the person. They found it easier to provide EOL care to a person who had an AHD in place, especially within indigenous communities, although this was also true with other communities. The benefits of attending the workshops were also profound among healthcare professionals who advocated for the provision of workshops by liaising with PCWA to deliver training for residents within retirement villages.

Participant 11, president of the residents committee at the retirement village where he and his wife live, said, ‘I have organised for PCWA to come and run a workshop. It turned out to be very, very popular and in fact, so many people signed up that they ran *2* workshops’. (field notes, June 10, 2024).

### Enacting the AHD

Substitute decision-makers also expressed confidence to enact the AHD knowing that they were acting to ensure the person’s values were respected and carried out. They also realised that it was not as difficult as they thought to follow someone’s wishes when they were documented. It gave them confidence that they were doing what the person wanted, therefore, they felt like a weight had been lifted off their shoulders.

. . .If there is an AHD, that helps us, because we can always go back to the document and see what the patient wants. But when there is no document, it is a whole complex process. (P21).

Another benefit of documenting preferences following attendance at the workshop was that completing the AHD helped families maintain relationships that may otherwise have been broken due to differing viewpoints about how to proceed with their loved one’s care.

And my son said to me, just as well he did that [completed AHD] because he said I would have fought him going at that stage. And so, I would have had cause to have a dispute with my sons, which I certainly wouldn’t have wanted to do. (P8)

### Case Studies

Table 2 below presents the stories from each of the participant groups about the benefit of attending ACP workshops and the impact the workshops had on care delivery.

**Table 2.** Case Studies of the Impact of Attending PCWA ACP Workshops.

Case study 1	Case study 2	Case study 3
Attending the workshop helped individuals see the need to have an AHD for themselves in place and to discuss their preferences with family/significant others.	Substitute decision-makers found the workshops beneficial.	Healthcare professionals found that when an AHD was in place it facilitated seamless care provision.
*‘So, I was with him [friend/neighbour]. And I was holding his neck, which was the only part of him that he could feel. But I was with him when he died. That would never have happened if I hadn’t have gone to the workshop and haven’t had my form checked. Cause we both now ha it in far more detailed now. . . Having seen what happened to him [friend/ neighbour]. I got my advance health directive, and I am absolutely certain, it went on to my health, my GP got it. I had given it to a couple of friends earlier. But ’,ve got the alerts in my home. Saying I have got one and where it is. I’ve got alerts everywhere because I live alone’. (P 5).* *‘My daughter has got my guardianship. And the other forms as well. So she, she has it. Yeah. And we’ve gone through it as the whole family. We’ve talked about this for a long time. So, we’re very open about this whole journey. And, I feel very confident. Sh’,s a health professional herself, so I feel I’m in safe hands, if you know what I mean. And that was the most important thing that I felt I got from it was still being able to totally understand the paperwork and then to come back to me and say, have you thought about or would you like to, and guiding you through that. That was just the best’. (P 9)* *“When you have actually completed the documents, you realise you haven’t really talked to your kids. Because the documents themselves talk about so many things that you would never have thought of, and the workshops prepare you for filling in the documents that then you realise it’s no good just to say, ‘Oh the kids know what I wan’, because they don’t and you and really this whole process is important to make sure that you can really have a conversation with the kids. . . our daughter or daughter-in-law is a radiographer down in the country. And so having a health professional in the family, meant the kids were accepting. ‘It wasn’t really a reaction at all, certainly not negative. They were saying, OK, that’s what you want. That’s grea’,. (P 11)*	*‘PCWA visited and provided us with quite a bit of information and support. PCWA also raised the subject of completing documents such as: Advanced Health Care Directive, Power of Attorney, and Power of Guardianship. . . That same day, the resident Doctor at the Hospice asked me about Colleen’s wishes in relation to her care. He also asked if she [wife] had an Advance Health Directive in place which I confirmed. . . the Doctors advised me that they would take good care of Colleen and would work and provide care, strictly in accordance with her advance health care plan [AHD]’, (P 3)* *‘I had an experience many years ago when my mother was very ill and my older brother made decisions without consulting mum or me – that caused so much tension that has’,t really resolved over these many years. I need to know what she [mother] wants now – you can’t un-revive someone if you do the wrong thing’. (P 13).* *‘I had to tell them what her wishes were. . .And then I had to make a decision within 12hours. I rang them all and told them that I was turning off a life support and gave them time by which they could come and say their goodbyes at the hospital together. They had to go one at a time because they had caused the disruption in the hospital. I was able to do that because the workshop trained me and gave me confidenc’,. (P 12)*	*‘It [PCWA ACP training] was really good. . . I was fairly new to palliative care but was expected to help patients understand AHD and the associated documents and, assist them [patients] fill out the paperwork. The training was really good because it developed my skills and understanding the document itself. So, I was able to provide good care. And also, knowing that I can reach out to PCWA at any time especially that I live in remote, that is even bette’,. (P21)* *‘’,ve attended a workshop on those things [ACP] because it’s useful in my role. . . And I do talk to our residents about the need to do one [AHD] and the impact of completing the document. And then sometimes there is a lack of awareness of what it actually means for them’. (P17)* *‘It [PCWA ACP workshop] was helpful. As a social worker, I’ve been in this role for three years and I think, you know, six months into the role, there was an expectation that we would support people to do their advanced health directives. I’m like, well! And how can I do that when I haven’t had training? I didn’t think it was appropriate. So, I asked to go and do some training, and I found it really helpful. It gave me context and obviously, it gave me more confidence in working with people and helping them understand what it is and helping them, you know, being able to identify what they wanted in terms of their values and then the life sustaining treatment. So, I’m glad I did it. I still think we need a bit mor’,. (P18)*

## Discussion

The purpose of this evaluation was to understand the longer-term impact of the PCWA ACP workshops and of having an AHD. To achieve that goal, the evaluation recruited and interviewed end-users of the workshops; individuals, primary carers, substitute decision-makers, and healthcare professionals. All of whom had attended community workshops led by PCWA but had different experiences according to their roles. The groups were interviewed to solicit information, not just about the benefits of the workshops in providing skills and information to the participants about the importance of ACP and preparing an AHD, but also how workshop participants utilised the gained skills and information going forward.

Data analysis in the evaluation of the long-term impact of workshops strongly suggests that participants found the workshops informative, beneficial, and consistent with previous evaluations^7,8^ and studies about ACP in the community^15^ The workshops enabled participants to gain insight not only into how to complete an AHD and other relevant documents such as the Enduring Power of Attorney (EPA) and EPG, but also how to have crucial conversations with family. Such workshops have the ability to provide an atmosphere for building confidence to discuss EOL matters.^16,17^ Studies also show that attending ACP workshops facilitates a change in attitude towards ACP and that, through interaction with other participants, they provide a good learning atmosphere.^17^ The workshops provided by PCWA certainly demonstrate the ability to change attitudes, boosting the confidence of individuals, carers, substitute decision-makers and healthcare professionals. EOL is not an easy topic,^18^ but PCWA has brought the WA community together to think and talk about something which is often perceived as a taboo subject.

Finding the words to communicate preferences in an AHD and share those preferences with family can be challenging. But when individuals felt confident that they had effectively conveyed their preferences, a sense of peace was attained which has been noted in previous research.^19^ One participant, whilst finding the workshop invaluable, had such difficulty in finding the words that she had suggested it would be helpful to video record her AHD. It should be noted that a person can express their EOL preferences in any format, including a video. These formats have legal standing (under common law), although would not be afforded the same legislative weight as a written AHD (which falls under statutory law). This could be reinforced in the workshops. However, it was clear from this evaluation findings that the value to the community provided by the workshops was immeasurable, as many participants shared the information with others.

The ripple effect of ordinary people sharing the positive outcomes of the workshops with their families, neighbours and even in parliament is evidence of the impact workshops had on participants. Studies such as Ref.,^19^ acknowledged the benefits of ACP discussions but conceded that with a lack of engagement from family/significant others, these conversations could be hampered. It is important therefore, to note that PCWA incorporates the ‘cards’ activity which many participants find useful in learning how to start a conversation. The cards activity involves using cue cards on palliative and EOL subjects, intended to help open conversations. These cards can be downloaded from the PCWA website. It was these skills and communication strategies imparted by PCWA that enabled participants to talk about the topic of ACP with people in their communities. While workshop participants were keen to advocate for the need to engage with ACP, the absence of GPs who are the primary providers of healthcare in this study, confirms the findings of previous studies. GPs have maintained their limited involvement in helping patients with the AHDs. Previous studies found that GPs did not actively promote and assist their patients with AHDs because of decisional conflict and ethical dilemmas when it comes to enacting the document,^20^ insufficient time to assist patients^21^ and having inadequate knowledge about AHD.^21,22^

Previous evaluations,^7,8^ have not followed up with substitute decision-makers or healthcare professionals to understand the impact of having an AHD when it comes to enacting the preferences on behalf of a loved one. The data clearly shows the positive impact completing the document has on the person carrying the responsibilities of decision-making. Participants who had experienced the death of a loved one, both with and without an AHD, there was a stark difference in personal grief and the risk of family fracture.

When combined with participation in the workshops, the evaluation found the substitute decision-makers had more confidence in their knowledge and ability to assert the preferences of their loved one. This empowering experience helped them, even as they reflected on a sad and difficult time. They felt comforted that they had done the best for their loved one which was a positive and life-affirming feeling even in their grief.

### Strengths and Limitations of the Evaluation

Recruiting for the evaluation proved difficult as seen by the proportion of participants when compared with contacts. This was also found in the last evaluation^8^ which may indicate that those that participated in the study were positively inclined towards the workshops thus, creating a bias in participant recruitment. Evaluation of the longer-term impact of the workshops for individuals is by its very nature difficult as they are only fully known after the person has passed away and the AHD has been. Yet it is these long-term consequences that have such importance for the health of those involved in EOL decision-making and care. The strengths of this evaluation are that individuals who attended workshops, carers and substitute decision-makers and healthcare professionals were represented, and their voices were heard. There was a lack of engagement however from GPs despite being invited after a referral from a substitute decision-maker. GPs are also key in ensuring individuals complete AHDs in a timely manner and key in enacting the AHD when required.^23^ Future long-term impact evaluations of the workshops may benefit from a prospective approach which would enable engagement with all those involved at the time. Although there was no participation by GPs in this study, the findings provide practical insights for practice, particularly in raising awareness of ACP and AHD, advocating for EOL decision-making and improving the quality of life and care in alignment with individual’s wishes. Additionally, the evaluation findings have the potential to influence and provides a valuable foundation for future cross-regional and cross-cultural research.

## Recommendations

Evaluation participants were keen for the workshops to continue because of the benefits they received from attending. The following were recommendations by participants:

1. PCWA workshops need to be scaled up to offer the service to more Western Australians.
2. Expand individualised service to help more people complete the documents.
3. Reinforce in the workshops the alternate methods for recording AHD, and the online resources available.
4. Offering GP’s tailored workshops to enable incorporating ACP discussions and documentation in primary health care.

The evaluating team recommended that PCWA consider a longitudinal prospective study of the workshops’ impact. This type of study would enable investigation of the relationship between outcomes and exposure to the workshops.

## Conclusion

This evaluation was undertaken to look at the long-term impact of the PCWA ACP workshops. Analysis revealed that participants considered the workshops to be important for individuals, families and communities in Western Australia. All participants supported PCWA continuing to offer the workshops as they were invaluable. Past workshop attendees were very keen for people in their communities to learn about and complete the AHD forms for themselves, as demonstrated when they encouraged family members and friends to attend subsequent workshops.

## Supplemental Material

sj-docx-2-inq-10.1177_00469580251371891 – Supplemental material for Advancing Person-Centred Care Through Palliative Care WA’s ACP Workshops: The Longer-Term Impact Supplemental material, sj-docx-2-inq-10.1177_00469580251371891 for Advancing Person-Centred Care Through Palliative Care WA’s ACP Workshops: The Longer-Term Impact by Gertrude G. Phiri, Emma Gunner, Philip Bafile and Davina Porock in INQUIRY: The Journal of Health Care Organization, Provision, and Financing

sj-pdf-1-inq-10.1177_00469580251371891 – Supplemental material for Advancing Person-Centred Care Through Palliative Care WA’s ACP Workshops: The Longer-Term Impact Supplemental material, sj-pdf-1-inq-10.1177_00469580251371891 for Advancing Person-Centred Care Through Palliative Care WA’s ACP Workshops: The Longer-Term Impact by Gertrude G. Phiri, Emma Gunner, Philip Bafile and Davina Porock in INQUIRY: The Journal of Health Care Organization, Provision, and Financing
