# Supplementary material for: Advancing Person-Centred Care Through Palliative Care WA’s ACP Workshops: The Longer-Term Impact
Source: Inquiry. 2025 Sep 28;62:00469580251371891. doi: 10.1177/00469580251371891 (PMC12477352; doi:10.1177/00469580251371891)
Supplement: sj-docx-2-inq-10.1177_00469580251371891 – Supplemental material for Advancing Person-Centred Care Through Palliative Care WA’s ACP Workshops: The Longer-Term Impact [file sj-docx-2-inq-10.1177_00469580251371891.docx]

**Guide Interview Questions**

**Interview questions for individual participants attending the workshops for personal benefit.**

1. Would you mind telling me your age?
2. What are your general impressions of the Advance Care Planning workshops?
3. Was the workshop useful and adequate in preparing you for end-of-life decision making?
4. Do you feel confident to put the concepts and knowledge gained into practice?
5. Do you know other people who are involved in providing end-of-life care who would like to participate in the study?

**Interview questions for family members/enduring guardian:**

1. Would you mind telling me your age?
2. Are you a family member who is providing care, an enduring guardian, or both?
3. How helpful was it to attend the workshop in making decisions on behalf of your family member?
4. Thinking about your family member:
5. Did/do they have an Advance Health Directive?
6. Did you talk to them about their wishes for end of life?
7. Please tell me how you have used the Advance Care Plan as part of the care of the family member.
8. How useful was/is the Advance Health Directive in your family member’s care?
9. How confident are/were you in following the Advance Care Plan as an enduring guardian?
10. What would you do differently if given another opportunity to make decisions on behalf of someone?
11. Do you know other people who are involved in providing end-of-life care who would like to participate in the study?

**Interview questions for health professionals and care staff associated with end-of-life care:**

1. Would you mind telling me your age?
2. What is your role in the team providing end-of-life care?
3. What training have you had on ACP/AHD?
4. Please describe your input into the care of the person with an Advance Care Plan (as identified by the family member/enduring guardian).
5. What was your experience of caring for someone who has documented or made known their Advance Care Plan decisions, preferences, and values (compared with those who have not)?
6. Do you know other people who are involved in providing end-of-life care who would like to participate in the study?
